# Chondrocytes, synoviocytes and dermal fibroblasts all express PH-20, a hyaluronidase active at neutral pH

**DOI:** 10.1186/ar1730

**Published:** 2005-04-04

**Authors:** Hafida El Hajjaji, Ada Asbury Cole, Daniel-Henri Manicourt

**Affiliations:** 1 Christian de Duve Institute of Cellular Pathology, Department of Biochemistry, Connective Tissue Group, Université Catholique de Louvain in Brussels, Brussels, Belgium; 2 Department of Biochemistry, Rush Medical College, Rush-Presbyterian-St. Luke's Medical Center, Chicago, IL, USA; 3 Department of Rheumatology, Saint Luke's University Hospital, Catholic University of Louvain in Brussels, Brussels, Belgium

## Abstract

Hyaluronan (HA), an important component of connective tissues, is highly metabolically active, but the mechanisms involved in its catabolism are still largely unknown. We hypothesized that a protein similar to sperm PH-20, the only mammalian hyaluronidase known to be active at neutral pH, could be expressed in connective tissue cells. An mRNA transcript similar to that of PH-20 was found in chondrocytes, synoviocytes, and dermal fibroblasts, and its levels were enhanced upon stimulation with IL-1. In cell layers extracted with Triton X-100 – but not with octylglucoside – and in culture media, a polyclonal antipeptide anti-PH-20 antibody identified protein bands with a molecular weight similar to that of sperm PH-20 (60 to 65 kDa) and exhibiting a hyaluronidase activity at neutral pH. Further, upon stimulation with IL-1, the amounts of the neutral-active hyaluronidase increased in both cell layers and culture media. These findings contribute potential important new insights into the biology of connective tissues. It is likely that PH-20 facilitates cell-receptor-mediated uptake of HA, while overexpression or uncontrolled expression of the enzyme can cause great havoc to connective tissues: not only does HA fragmentation compromise the structural integrity of tissues, but also the HA fragments generated are highly angiogenic and are potent inducers of proinflammatory cytokines. On the other hand, the enzyme activity may account for the progressive depletion of HA seen in osteoarthritis cartilage, a depletion that is believed to play an important role in the apparent irreversibility of this disease process.

## Introduction

Hyaluronan (HA), a linear, megadalton glycosaminoglycan, has important biological and structural functions [1]. First, by interacting with transmembrane proteins, such as CD44 and other members of the heterogeneous group of proteins termed hyaladherins, HA initiates signaling pathways and contributes to the formation of the pericellular matrix that prevents direct contact between cells and protects them against attack from viruses, bacteria, and immune cells. Second, in the extracellular matrix further removed from the cell and in the basement membrane, the hydrophilic HA network not only gives turgor pressure and resilience, but also functions as a scaffold about which other macromolecules associate and orient themselves [2-4]. Within the abundant extracellular matrix of articular cartilage, the long, filamentous HA molecules form the backbone upon which the viscoelastic aggrecan molecules align to form aggregates, a supramolecular organization that immobilizes aggrecans at very high concentrations within the collagen network, thereby providing remarkable biomechanical properties to the articular tissue [5]. Third, in its unaggregated form, HA is the major macromolecular species in synovial fluid, being thereby responsible for the viscoelastic properties of what is otherwise a simple plasma diffusate [6].

On the other hand, because HA degradation products may interact with various cells and initiate a program of gene expression leading to cell proliferation, migration, or activation [3,4,7], these products exhibit biological functions that are quite distinct from those of the native, high-molecular-weight polymer. Thus, by stimulating the proliferation and migration of vascular endothelial cells via multiple signaling pathways, HA fragments induce angiogenesis, whereas high-molecular-weight HA inhibits angiogenesis [8,9]. Studies *in vitro *have also indicated that HA fragments similar in size to that of fragmented HA molecules found *in vivo *in inflammatory sites induce the expression of inflammatory genes in dendritic cells, macrophages, eosinophils, and certain epithelial cells [7]; this effect is in contrast to that of high-molecular-weight HA molecules, which inhibit the production of IL-1, prostaglandin E_2_, and matrix metalloproteinases [10-12]. Further, HA depolymerization, such as occurs in osteoarthritis and other arthritides, compromises the biomechanical properties of diarthrodial joints and thereby contributes to joint destruction. Indeed, the synovial fluid of diseased joints contains smaller HA molecules, which dramatically reduce the lubricating properties of the joint fluid [13]. On the other hand, upon stimulation with IL-1, the HA molecules in articular cartilage explants are fragmented and lost into the conditioned medium [14], a finding which implies that in the presence of this cytokine the viscoelastic aggrecan molecules are no longer firmly entrapped within the collagen network of the articular tissue.

Since the fragmentation of HA compromises the integrity of tissues, it is obviously important to investigate the mechanisms involved in the production of its fragments. Although oxygen-derived free radicals are known to fragment HA randomly [15], one cannot exclude the possible role of hyaluronidases, because mammalian hyaluronidases, in contrast to bacterial ones, yield a heterogeneous mixture of oligosaccharides and HA fragments of various sizes.

Thus far, the only human hyaluronidase known to be active at neutral pH is the sperm adhesion molecule 1, also termed PH-20, which enables the sperm to penetrate the HA-rich cumulus oophorus [16,17]. Therefore, and although PH-20 mRNA has been detected only in testis, mouse kidneys, some cancer cell lines, and fetal and placenta cDNA libraries [18-21], we hypothesized that connective tissue cells may express a hyaluronidase similar to the one present on sperm. Herein we provide evidence that, at both the mRNA and protein levels, fibroblasts, chondrocytes, and synoviocytes all express a neutral, active hyaluronidase similar to sperm PH-20.

## Materials and methods

### Culture media and reagents

The following reagents were used: DMEM, FCS, penicillin, streptomycin, the PCR TOPO vector, and the SuperScript Reverse Transcriptase (Invitrogen, Merelbeke, Belgium); the Complete Protease Inhibition Cocktail, the Tripure isolation kit, collagenase P, *n*-octylthioglucoside, recombinant human IL-1β (IL-1; specific activity 5 × 10^7 ^units/mg) (Roche Applied Science, Brussels, Belgium); radiolabelled precursors, Hybond-N^+ ^membranes, CNBr-activated Sepharose, EAHSepharose, Megaprime DNA labeling system, and restriction enzymes (Amersham, Roosendaal, the Netherlands); standards and reagents for protein electrophoresis (Bio-Rad laboratories, Nazareth, Belgium); Pierce BCA protein reagent, Pierce Immuno-Pure Gentle Ag/Ab Elution buffer, and Clontech Advantage PCR cDNA mix kit (Perbio Science, Erembodegem, Belgium); DNA molecular-weight markers and custom-made primers (Eurogentec, Seraing, Belgium); the pGEM-T Easy Vector kit (Promega Benelux, Leiden, the Netherlands); Qiagen kits for plasmid purification (Westburg, Venlo, the Netherlands); and BSA, high-purity fraction V, from Calbiochem (VWR International, Leuven, Belgium). Unless specified, all other chemicals used were of analytical grade and obtained from Sigma-Aldrich-Fluka (Bornem, Belgium).

### Tissue acquisition and cell culture

Human chondrosarcoma cells (SW 1353) were purchased from the American Tissue Culture Collection (LGC Promochem, Teddington, UK). Tissue sampling was conducted in accordance with the rules and regulations of the Saint Luc University Hospital ethics committee (UCL, Brussels). Foreskin was obtained from newborn babies undergoing surgery for phymosis. Human articular cartilage and synovium were harvested from patients undergoing either leg amputation or arthroplasty of the knee joint. Dermal fibroblasts and synoviocytes were isolated by sequential enzymatic digestion with 0.15% collagenase P and 0.03% trypsin, and the cells were used routinely at passage numbers 4 to 7. Chondrocytes were isolated [22] and used at passage number 2 or 3.

Cells were cultured in DMEM supplemented with 10% heat-inactivated FCS, penicillin (50 units/ml), and streptomycin (50 μg/ml). At 2 days before cell sampling and/or cell stimulation by cytokines, FCS was replaced by ITS (insulin, transferrin, and sodium selenite) and 0.5% BSA. In contrast to other tested preparations, the BSA (fraction V, high purity) that we routinely used from Calbiochem did not contain any hyaluronidase activity as assessed by HA substrate gel electrophoresis.

At the end of the culture period, conditioned media were removed and enriched with Triton X-100 (1% final concentration) and a complete protease inhibitor cocktail before further processing. The Tripure solution was then directly added to the culture dish kept on ice to extract the total cellular RNA. In parallel experiments, 50 mM sodium phosphate, pH 7.4, containing the cocktail of protease inhibitors as well as either 1% Triton X-100 or 20 mM octylthioglucoside was added to culture dishes kept on ice to extract proteins from cell layers. Adherent cells were scraped off with a rubber scraper and further incubated with the extracting solution on ice for 15 min with intermittent vortexings. At the end of the incubation period, the solution was centrifuged and the supernatant was either used immediately or stored at -20°C in glycerol until use. The pellet represented a very small proportion of the original cells.

### Reverse transcriptase polymerase chain reaction

For reverse transcription reactions, about 250 ng of total RNA was converted into first-strand cDNA using SuperScript Reverse Transcriptase in accordance with the manufacturer's instructions, in a final volume of 20 μl. The target cDNA was then amplified by using the Advantage cDNA PCR kit, a reaction that included 2 μl of the cDNA synthesis reaction. After 35 cycles at 94°C for 30 s, 64°C for 1 min, and 72°C for 5 min, amplification products were detected by electrophoresis in 1% agarose gel containing 0.5 μg/ml ethidium bromide, into which 10 μl of sample per lane was injected. DNA molecular-weight markers were included on each gel for sizing.

Comparison between the sequence of testis PH-20 cDNA (GenBank accession number S67798) and the sequence of chromosome 7q31.3 (4676254 to 4710990) reveals that sperm PH-20 cDNA contains apparently four exons, the coding sequence starting in exon 2 and ending in exon 4. Therefore, to avoid genomic DNA contamination, two sense primers in exon 2 and two antisense primers in exon 4 were chosen. The first set of primers (primers A) consisted of 5'-CCA-TGTTGCTTGACTCTGAATTTCA-3' (oligo sense) and 5'-CCGAACTCGATTGCGCACATAGAGT-3' (oligo antisense), with an expected RT-PCR product of 759 bp. The second set of primers (primers B) consisted of 5'-GCCTGGAATGCCCCAAGTGA-3' (oligo sense) and 5'-TCCTTGCTCCTGGCAAAGCAC-3' (oligo antisense), with an expected RT-PCR product of 1,000 bp. A third set of primers (primers C) was chosen both in exon 4: 5'-TGCTTTGCCAGGAGCAAGGA-3' (sense primer) and 5'-CCTGCGCAATTACAAACT-CGCTACA-3' (oligo antisense), with an expected RT-PCR product of 403 bp. Primers used for the housekeeping gene β-actin were 5'-TGATGGTGGGCATGGGTCAG-3' (oligo sense) and 5'-TCTTCTCGCGGTTGGCCTTG-3' (oligo antisense), with an expected RT-PCR product of 226 bp.

### Cloning and sequencing of PCR products

Aliquots of RT-PCR products were cloned in the pGEM-T Easy Vector. Recombinant plasmids were isolated and sequenced by using the Big Dye Terminator Cycle sequencing kit from Applied Biosystems (Lennik, Belgium) and an automated sequencer (ABI Prism model 310).

### Northern blot analysis

As the 1,000-bp RT-PCR product amplified by PH-20 primers B does not contain any *Eco*RI restriction sites, this product was cloned into the PCR TOPO vector that exhibits *Eco*RI sites flanking the PCR product insertion site. The approximately 1,000-bp *Eco*RI restriction fragment was radiolabelled with [α-^32^P]dCTP by random priming (Megaprime system) and used as a PH-20 probe.

Intact RNA samples (25 μg) were subjected to electrophoresis in 1% agarose/formaldehyde gels and transferred by capillarity to Hybond-N^+ ^membranes prior to fixation by UV cross-linking. After a prehybridization step for 4 hours in 6 × SSC (1 × SSC is 0.15 M NaCl, 0.015 M sodium citrate, pH 7.0), 5 × Denhardt's solution, 0.5% SDS, and 20 μg/ml denatured Salmon sperm DNA (Invitrogen, Merelbeke, Belgium), the membranes were hybridized overnight at 64°C in a similar solution containing either the PH-20 or the actin probe. At the end of the hybridization procedure, membranes were washed at 64°C (2 × SCC, 3 × 10 min; 2 × SSC/0.1% SDS, 1 × 10 min; 2 × SSC, 1 × 10 min; 0.2 × SSC, 1 × 10 min), dried, and then exposed to x-ray film for 12 hours (β-actin) or 48 hours (PH-20).

In separate experiments, 20 μg of total RNA from cells either nonstimulated or stimulated with IL-1 at a concentration of 5 or 10 ng/ml were subjected to northern blotting as stated above and the dried membranes were exposed to a PhosphorImager screen (Molecular Dynamics, Sunnyvale, CA, USA) for the detection and quantification of the radioactive signals. As the 1.4-kb transcript represented approximately 10% of the total radioactivity, quantification was restricted to the 2.4-kb transcript. Results obtained for stimulated cells were normalized with values obtained for the corresponding unstimulated control cells, to which a value of 1 arbitrary unit was assigned. In each set of experiments, three Petri dishes (79 cm^2 ^each) containing cells at near confluence were used for each condition. Data represent the means ± standard deviations obtained for five different sets of experiments.

### Antibody production and PH-20 purification

In accordance with the sequence of human PH-20 (Swissprot: locus HYAP_HUMAN, accession P38567), the peptide NH2-CAR NWK PKD VYK NRS I-CONH_2 _(amino acids 155 to 169) was chosen. The sequence of its first 10 amino acids is also present in bovine PH-20. Further comparison between the sequence of the immunizing peptide and that of other known human proteins ('blast', National Institure of Health) also reveals that both hyaluronidases 2 [23] and 4 [20] exhibit two stretches of three to four identical amino acids separated by different amino acids, whereas hyaluronidase 1 [24] contains three stretches of two identical amino acids. After synthesis, the 15-mer oligopeptide was coupled to keyhole limpet hemocyanin and injected into rabbits for immunization. After the peptide had been coupled to EAHSepharose (efficiency 98%), the peptide–gel complex was incubated with the serum and then washed extensively with Tris-buffered saline. The specific antibodies were eluted with 1.5 M guanidine hydrochloride and stored in PBS containing 0.01% sodium azide and 1% BSA.

Purified antibodies were covalently coupled to CNBr-activated Sepharose in accordance with the manufacturer's instructions (3 mg IgG per gram dry gel). Cell-layer extracts and conditioned media containing a cocktail of protease inhibitors were applied to columns packed with the immunoaffinity gel, which was then washed with 20 column volumes of PBS containing 0.1% Triton X-100 before eluting the bond proteins with either 50 mM glycine–HCl, pH 3.0, or the Pierce Immuno-Pure 'gentle' Ag/Ab elution buffer. Fractions eluted with the glycine–HCl buffer were neutralized immediately by adding 0.1 volume of 1 M Tris–HCl, pH 8.0. The BCA assay was used to determine protein concentrations.

The immunoaffinity gel was also used to estimate the changes in the amounts of PH-20 proteins present in cell layers and conditioned media of the three cell lines upon stimulation with IL-1. For each cell line and in each experiment, three Petri dishes (79 cm^2 ^each) containing cells at near confluence were used for each condition. The data reported are means ± standard deviations obtained for three to five sets of experiments.

### Sodium dodecyl sulfate–polyacrylamide gel electrophoresis

After being concentrated by ultrafiltration, proteins eluted from the immunoaffinity gel were characterized by Tricine (*N*-tris [(hydroxymethyl)methyl]glycine) SDS–PAGE in accordance with the procedure of Schagger and von Jagow [25]. Gels were fixed in 50% methanol and 10% acetic acid before being stained with 0.025% Serva Blue G in 10% acetic acid.

### Assays for hyaluronidase activity

In a first set of studies, hyaluronidase activity was detected by HA substrate gel electrophoresis [26]. Briefly, after electrophoresis at 4°C (20 mA/gel), gels were washed twice for 20 min in 2.5% Triton X-100 before overnight incubation in 0.15 M sodium chloride, 0.5 mM calcium chloride, 7 mM 1,4-saccharolactone buffered with either 0.1 M acetate, pH 4.5, or 0.1 M Mes (2-(N-Morpholino)ethane sulfonic acid), pH 6.5. When required, apigenin dissolved in dimethylsulfoxide was added to the incubation solution to give a final concentration of 5 μg/ml. Control gels were incubated with a similar volume of dimethylsulfoxide. After incubation, gels were washed twice for 30 min in water and further incubated at 37°C with proteinase K to remove proteins that may interfere with gel staining with alcian blue. The presence of bands without staining on the blue background of undegraded HA indicates hyaluronidase activity.

Hyaluronidase activity was also assessed by using high-molecular-weight radiolabelled HA (5 × 10^6 ^dpm (disintegrations per minute) per milligram). To generate this substrate, preconfluent synoviocytes were incubated in the presence of 2-acetamido-2-deoxy-D-glucurono-1,5-lactone (50 mM) and tritium-labelled glucosamine (50 μCi/ml); after digestion with proteinase K and precipitation in ethanol, the glycosaminoglycans were passed through a DEAESepharose column equilibrated in 50 mM pyridine, pH 5.5 (buffer A), and the high-molecular-weight tritium-labelled HA molecules were eluted at a NaCl concentration of 0.4 M. Assays were conducted in duplicate as follows: specimens and known relative turbidity-reducing units (rTRUs) of testicular hyaluronidase were incubated with purified tritium-labelled HA molecules (5 to 8 μg, 4 × 10^4 ^dpm) in either 0.1 M Hepes (4-(2-hydroxyethyl)-1-piperazine-ethanesulfonic acid) (pH 7 to 8), 0.1 M Mops (3-(*N*-morpholino)propanesulfonic acid) (pH 6.5 to 7.5), 0.1 M Mes (pH 5.5 to 6.5), or 0.1 M acetate (pH 4.5 to 5.5) containing a final concentration of 0.15 M NaCl, 7 mM 1,4-saccharolactone, and 1% Triton X-100. After incubations, specimens were heated at 95°C for 5 min, diluted with 10 volumes of buffer A, and applied on 1 ml DEAESepharose gel equilibrated in the same buffer. Gels were then washed with 10 volumes of buffer A before being eluted step by step with increasing amounts of NaCl dissolved in the same buffer: 10 volumes of 0.2 M NaCl for step 1; 10 volumes of 0.3 M NaCl for step 2; 10 volumes of 0.4 M NaCl for step 3, and 3 volumes of 0.8 M NaCl for step 4. More than 95% of the radioactivity applied onto the gel was recovered in steps 1 to 3. HA disaccharides produced by chondroitinase ABC elute at 0.2 M NaCl, the HA tetrasaccharides and hexasaccharides produced by testicular hyaluronidase elute at 0.2 and 0.3 M NaCl, and the intact HA molecules elute at 0.4 M NaCl. For amounts of testicular hyaluronidase ranging between 0.005 and 0.05 rTRUs, there is a highly statistically significant linear correlation (*r *= 0.9) between, on the one hand, the enzyme activity and, on the other hand, the % of radioactivity recovered in steps 2 and 3 (range 10 to 45%). The intra-assay and interassay variations are less than 5 and 10%, respectively.

## Results

### mRNA for PH-20 is present in chondrocytes and other connective tissue cell lines

**Figure 1:**
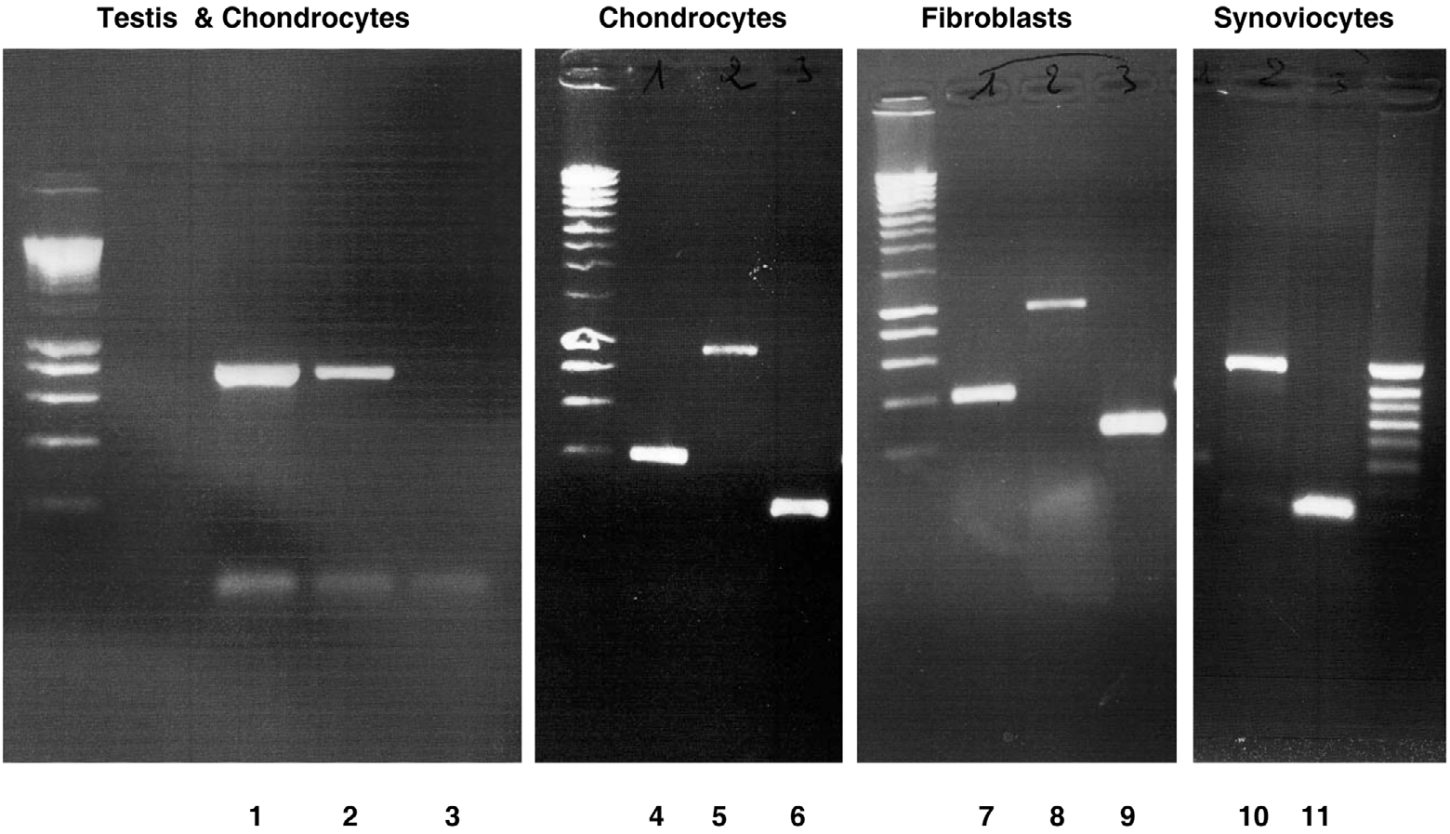
Gene-specific RT-PCR survey of mRNA for the hyaluronidase PH-20 in human connective tissue cells. Three sets of primers amplified the expected product: 759 base pairs (bp) with primers A (lane 1, testis; lane B, chondrocytes; lane 3, negative control), 1,000 bp with primers B (lanes 5, 8, and 10), and 403 bp with primers C (lanes 4 and 7). Primers for actin also amplified an expected product of 226 bp (lanes 6, 9, and 11).

RT-PCR was used to investigate whether PH-20 mRNA was present in human chondrocytes, fibroblasts, or synoviocytes and also in a chondrosarcoma cell line (Fig. 1). Primers A amplified an expected RT-PCR product of 759 bp in both testis (lane 1) and chondrocytes (lane 2), but the amplified product was more abundant in testis than in chondrocytes. RT-PCR products of expected size were also amplified by using either PH-20 primers B (1,000 bp: lanes 5, 8, and 10), PH-20 primers C (403 bp; lanes 4 and 7) or actin primers (226 bp; lanes 6, 9, and 11). In all the cell lines tested, the band obtained for the housekeeping gene β-actin was stronger than that observed for PH-20.

After purification, these products were ligated into the pGEM-T vector and sequenced on both strands. The sequence obtained for each cell line was identical to the sequence of human testis PH-20.

**Figure 2:**
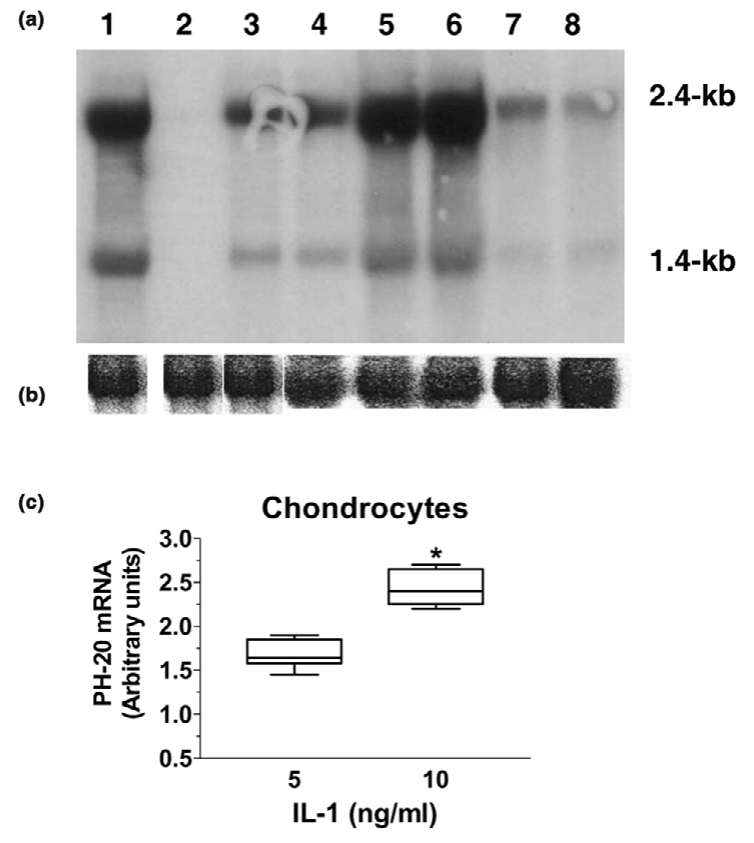
Northern blot analysis of mRNA from various connective tissue cell lines. **(a) **A radiolabelled PH-20 cDNA probe was hybridized to a northern blot containing 25 μg/lane of total RNA from (1) testis (positive control); (2) liver (negative control); (3) synoviocytes; (4) chondrocytes; (5) chondrocytes stimulated with 5 ng/ml of IL-1; (6) chondrocytes stimulated with 10 ng/ml of IL-1; (7) fibroblasts; (8) a chondrosarcoma cell line. **(b) **For loading control, the same blot was stripped and hybridized with a β-actin probe. **(c) **Column box–whisker plot showing the relative increase of the PH-20 2.4-kilobase transcript in chondrocytes stimulated with IL-1 at two concentrations (*n *= 5; *P = 0.0004 by paired *t*-test).

Northern blotting was used to further characterize the PH-20 mRNA (Fig. 2a). As observed in testis (lane 1), the probe detected a strong 2.4-kb mRNA band in synoviocytes (lane 3), chondrocytes (lane 4), fibroblasts (lane 7), and a chondrosarcoma cell line (lane 8), whereas no signal was detected in the RNA extracted from liver (lane 2), a tissue reported as not expressing PH-20 [27]. The PH-20 probe also detected a fainter, 1.4-kb band whose abundance was somewhat related to the abundance of the major 2.4-kb band. Thus far, it is not clear whether the smaller transcript indicates alternative splicing of PH-20 mRNA or reflects the existence of another potential hyaluronidase. This notwithstanding, it is worth noting that the signal corresponding to the other known hyaluronidases (1, 2, 3, and 4) is greater than 2 kb [20] and that PH-20 RT-PCR conducted either with sense primer located in exon 1 and antisense primer located in exon 3 or with sense primer located in exon 2 and antisense primer in exon 4 always gave a product of expected size (not shown).

Because HA depolymerization is consistently observed in inflammatory sites [7], and as the HA molecules present in articular cartilage explants are fragmented and lost into the conditioned medium upon stimulation with IL-1 [14], we examined the effect of this proinflammatory cytokine on PH-20 mRNA levels in chondrocytes: the band intensity was enhanced at IL-1 concentrations of both 5 ng/ml (lane 5) and 10 ng/ml (lane 6). The intensity of the 2.4-kb transcript was semiquantified by using the PhosphoImager (Fig. 2c): the intensities increased (mean ± standard deviation) by a factor of 1.7 ± 0.2 (*n *= 5) at 5 ng/ml IL-1 and 2.4 ± 0.3 (*n *= 5; *P *= 0.0004) at 10 ng/ml IL-1. Concentration of 10 ng/ml.

### Evidence for the presence of the PH-20 protein in cell-associated extracts and conditioned media

**Figure 3:**
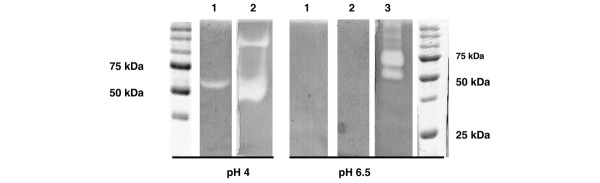
Hyaluronan (HA) substrate gel electrophoresis of fibroblast cell layers extracted with octylglucoside. After electrophoresis, gels were incubated at 37°C for 16 hours at pH 4 or pH 6.5 before being treated with proteinase K and stained with alcian blue. Lane 1, fibroblast cell layers extracted with octylglucoside; lane 2, a preparation of liver lysosomes; lane 3, a commercial preparation of bovine testicular hyaluronidase. Standards were Precision Plus Protein standards from Bio-Rad, with major bands at 75 and 50 kDa).

Because, thus far, chondrocyte and fibroblast cell layers extracted with octylglucoside have been reported to contain a hyaluronidase activity that cannot be detected above pH 5 [22,28], a preparation of fibroblast cell layers extracted with octylglucoside was subjected to HA substrate gel electrophoresis (Fig. 3). As observed by Stair-Nawy and colleagues [28], the fibroblast preparation (lanes 1) exhibited a clear-cut band of activity with an apparent molecular weight of approximately 55 kDa at pH 4, but no activity was detected at pH 6.5. Likewise, a preparation of liver lysosomes used as positive control (lanes 2) was very active at pH 4, with a major band at approximately 50 kDa and a minor band at approximately 120 kDa, but was totally inactive at pH 6.5. On the other hand, a commercial preparation of bovine testicular hyaluronidase (lane 3) exhibited several bands of activity at near neutral pH. Therefore, we hypothesized that octylglucoside was apparently unable to extract from cell layers a neutral-active hyaluronidase, or at least not in amounts sufficient to be detected by HA substrate gel electrophoresis.

**Figure 4:**
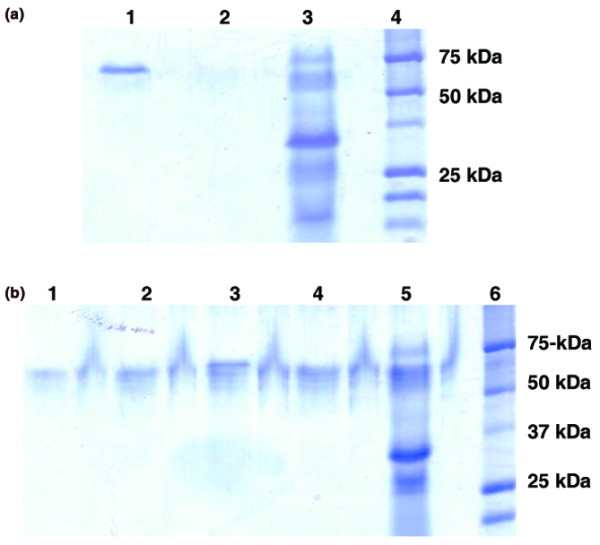
Tricine SDS-PAGE (T-SDS-PAGE) of proteins purified with the anti-PH-20-antibodies–Sepharose gel. **(a) **Triton X-100-extracted (lane 1) and octylglucoside-extracted (lane 2) fibroblast cell layers were allowed to interact with the immunoaffinity gel, and fractions eluted with the glycine–HCl buffer were analyzed using T-SDS-PAGE. Lane 3, commercial preparation of bovine testis hyaluronidase; lane 4, Precision Plus Protein standards from Bio-Rad. A strong band was detected in the Triton X-100 extracts, whereas a faint band was barely detected in the octylglucoside extracts. **(b) **Triton X-100-extracted chondrocyte cell layers and their conditioned media were applied to immunoaffinity columns. Samples eluted with the glycine–HCl buffer were subjected to T-SDS PAGE. Unstimulated chondrocyte cell layers (lane 3; 1.4 μg) and their conditioned medium (lane 1; 0.8 μg). Cell layers of chondrocytes stimulated with 5 ng/ml of IL-1 (lane 4; 1.2 μg) and their conditioned medium (lane 2; 1 μg). Lane 5, commercial preparation of bovine testis PH-20; lane 6, Precision Plus Protein standards from Bio-Rad. Several bands ranging from approximately 60 to approximately 65 kDa can be identified in each specimen.

Because obviously fibroblasts expressed PH-20 at the mRNA level, we used the anti-PH-20 antibodies to test whether these cells expressed the neutral-active hyaluronidase at the protein level. After having extracted fibroblast cell layers with either octylglucoside or Triton X-100, a detergent used to solubilize sperm PH-20 [17], specimens from both extracts (approximately 3 mg proteins) were applied to columns packed with anti-PH-20-antibodies–Sepharose. Fractions eluted with the glycine–HCl buffer were concentrated by ultracentrifugation and analyzed using SDS-PAGE (Fig. 4a). A strong band (lane 1) with the apparent molecular weight of 65 kDa reported for sperm PH-20 [29] was present in specimens extracted with Triton X-100, whereas a faint band with a similar molecular weight could be barely detected in specimens extracted with octylglucoside (lane 2). These results confirmed that a substantial population of proteins recognized by anti-PH-20 antibodies and having a molecular weight similar to that of the sperm hyaluronidase are extractable by Triton X-100 but not by octylglucoside [17,29].

In parallel experiments, chondrocytes were cultured in the absence and in the presence of IL-1 (5 ng/ml). Cell layers extracted with Triton X-100 as well as their corresponding culture media were allowed to interact with the immunoaffinity gel, and proteins eluted with the glycine–HCl buffer were subjected to SDS-PAGE (Fig. 4b). Specimens from unstimulated and stimulated conditioned media (lanes 1 and 2, respectively) as well as extracts from unstimulated and stimulated cell layers (lanes 3 and 4, respectively) all exhibited several bands ranging from approximately 60 to 65 kDa. This range of molecular weights has been observed for human and macaque sperm PH-20 and is thought to be related to the glycoprotein structure of PH-20 [29,30]. Further, although the commercial preparation of bovine testis hyaluronidase (lane 5) contained a major band of approximately 33 kDa and a well-defined band at approximately 69 kDa, and a diffuse pattern of bands ranging from 60 to 65 kDa could also be observed. SDS-PAGE of specimens from cell-layer extracts and conditioned media of synoviocytes gave similar results (not shown).

**Figure 5:**
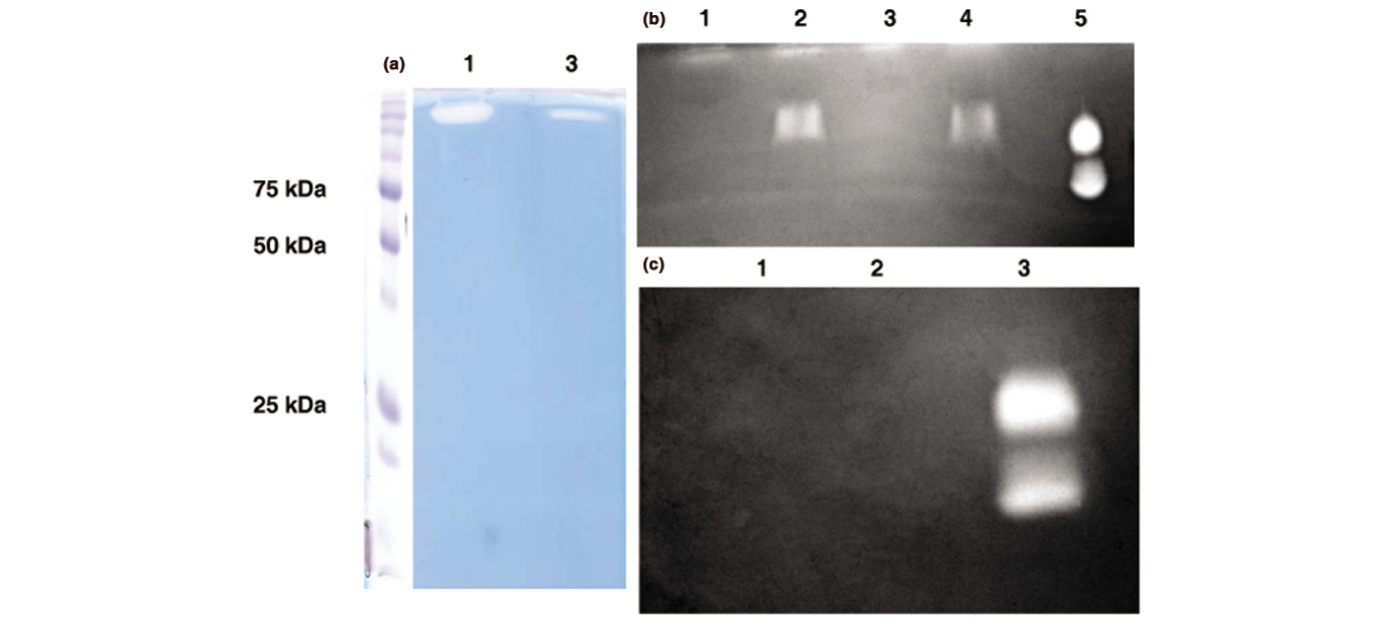
Hyaluronan (HA) substrate gel electrophoresis of chondrocyte cell layers extracted with Triton X-100. **(a) **Specimens of fibroblast cell layers extracted with Triton X-100 (3 mg as total protein) were applied to an anti-PH-20-antibody Sepharose gel and eluted with either the ImmunoPure Gentle Ag/Ab buffer (lane 1) or the glycine–HCl buffer (lane 3) were subjected to HA substrate gel electrophoresis. A band of activity with an unexpected molecular weight (>200 kDa) was observed in both specimens. **(b) **When HA oligosaccharides were added to specimens eluted with either the 'gentle' buffer or the glycine–HCl buffer, the band of activity appeared at the expected molecular weight: 'gentle' buffer-eluted specimens without (lane 1) and with (lane 2) HA oligosaccharides; glycine–HCl buffer-eluted specimens without (lane 3) and with (lane 4) HA oligosaccharides; lane 5, commercial preparation of testicular hyaluronidase. Similar results were obtained with specimens from fibroblast and synoviocyte cell layers. **(c) **No hyaluronidase activity was detected in the HA oligosaccharide preparation (lane 1) or in the 'gentle' buffer preparation (lane 2); lane 3, commercial preparation of testicular hyaluronidase.

**Figure 6:**
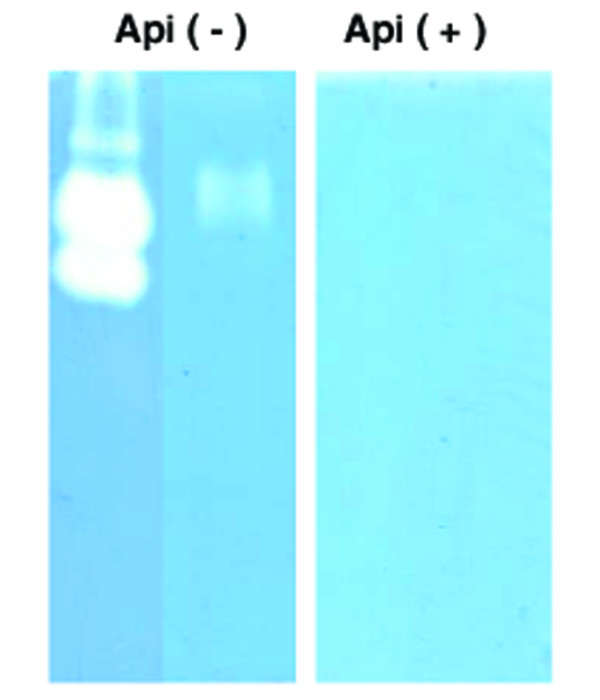
Apigenin inhibits the hyaluronidase activity of immunopurified proteins. The hyaluronidase activity present in a commercial preparation of testicular hyaluronidase (Api(-), left lane) as well the hyaluronidase activity purified from cell layers with the immunoaffinity gel (Api(-), right lane), both disappeared when HA substrate gels were incubated in the presence of apigenin (Api(+)).

The hyaluronidase activity of the proteins recognized by PH-20 antibodies was examined next. Proteins eluted from the immunoaffinity gel either with the rather harsh glycine–HCl buffer or with the milder Immuno-Pure^® ^Gentle elution buffer were subjected to HA substrate gel electrophoresis at pH 6.5 (Fig. 5). Surprisingly, specimens eluted with the gentle buffer (Fig. 5a, lane 1) and those eluted with the glycine–HCl buffer (Fig. 5a, lane 3) both exhibited a single band of activity with an apparent molecular weight of above 200 kDa. Because specimens were not heat-denatured and because primate sperm PH-20 has a HA binding domain that is distinct from the hyaluronidase domain [17,31], we hypothesized that the binding of high-molecular-weight HA molecules to the PH-20 molecules may hamper the protein's movement to its expected location. Therefore, because HA oligosaccharides are known to dissociate the non-link-stabilized aggrecan molecules from their high-molecular-weight HA backbone [32], we accordingly prepared HA oligosaccharides varying in length from 10 to about 30 monosaccharides, which were then allowed to interact with specimens (about 1 mg of HA oligosaccharides/mg proteins) before being loaded into the gel. When this procedure was used (Fig. 5b), the band of activity switched from the apparent molecular weight of >200 kDa to the expected apparent molecular weight of 60 to 65 kDa in specimens eluted with the gentle buffer (lanes 1 and 2) and also in specimens eluted with the glycine–HCl buffer (lanes 3 and 4). We were unable to detect any hyaluronidase activity (Fig. 5c) in the HA oligosaccharide preparation (lane 1) or the gentle buffer (lane 2). On the other hand, the hyaluronidase activity was stronger in specimens eluted with the gentle buffer (Fig. 5a, lane 1, and 5b, lane 2) than in specimens eluted with the glycine buffer (Fig. 5a, lane 3, and 5b, lane 4), an observation suggesting that the glycine buffer is likely to be harmful to the hyaluronidase activity of the eluted proteins. Further, both the hyaluronidase activity eluted from the immunoaffinity gel and that of the preparation of bovine testicular hyaluronidase disappeared when the HA substrate gels were incubated in the presence of apigenin (Fig. 6), a well-known inhibitor of hyaluronidase [33]. This observation and the presence of saccharolactone in the incubation buffer strongly suggest that HA degradation reflects the hyaluronidase activity alone rather than the combined action of hyaluronidase and exoglycosidases.

### Further characterization of the hyaluronidase activity present in cell-layer extracts and conditioned media

**Figure 7:**
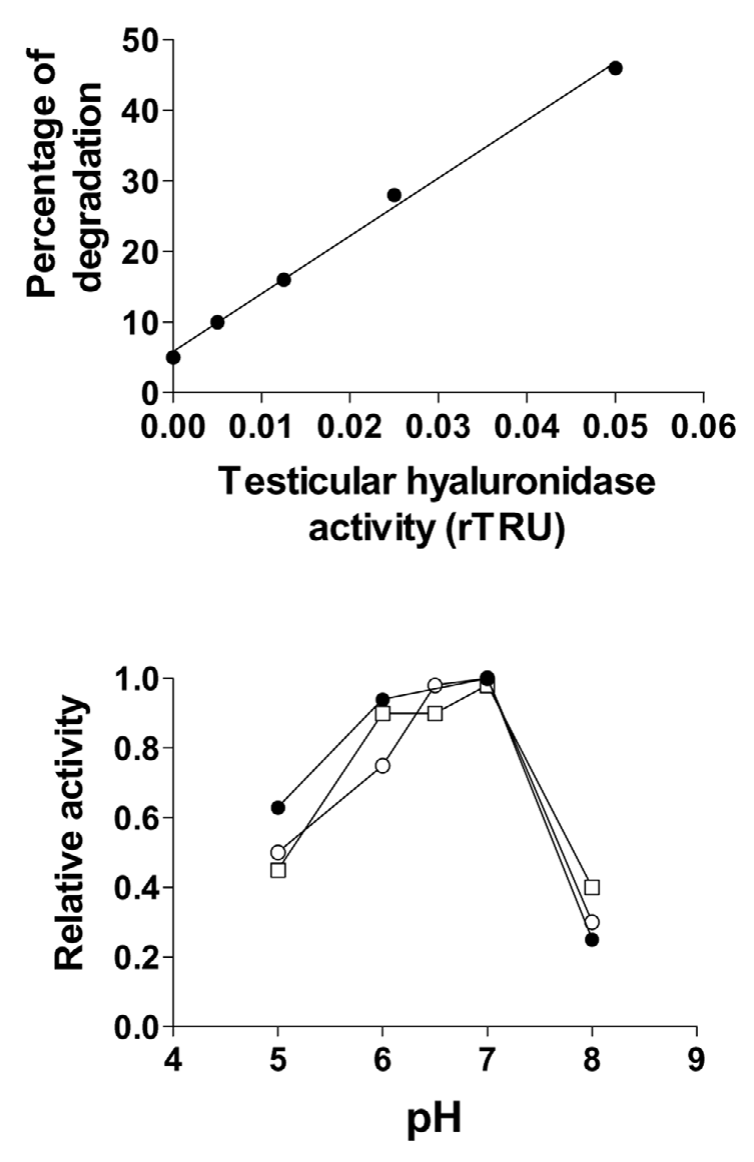
Detection of hyaluronidase activity by a radiolabelled hyaluronan (HA) substrate assay. **(a)**Standard curve between the relative percentage of degraded HA molecules and the relative turbidity-reducing units (rTRUs) of hyaluronidase. **(b) **pH profile of the activity of proteins immunoprecipitated from chondrocyte-layer extracts (open squares), chondrocyte-conditioned media (open circles) and a commercial preparation of bovine testicular hyaluronidase (closed circles). Samples were analyzed in triplicate and activity was expressed as a percentage of maximal activity (= relative activity). A similar pH profile was obtained with specimens from synoviocyte-layer extracts and conditioned media.

The pH profile of the hyaluronidase immunoprecipitated from cell-layer-associated extracts and conditioned media was further examined using the radiolabelled HA substrate assay, a procedure that can detect with reliability an enzymatic activity ranging between 0.005 and 0.05 rTRUs (Fig. 7, upper panel) and that is thus quite a bit more sensitive than HA substrate gel electrophoresis. Although elution with a very acidic pH seems to reduce the hyaluronidase activity of the eluted proteins as assessed by HA substrate gel electrophoresis, preparations of cell layer extracts and conditioned media as well as a commercial preparation of testicular hyaluronidase were eluted from the immunoaffinity gel with the glycine–HCl buffer, because the composition of the gentle elution buffer from Pierce was not known. Eluted proteins were then diluted to be in the linear range of the assay before being incubated for 16 hours at 37°C in appropriate buffers whose pHs were systematically adjusted for a temperature of 37°C. Because the stock of tritium-labelled HA substrate was in 0.4 M NaCl, the assay had to be conducted in 0.10 M NaCl, although this salt concentration has been reported to decrease the apparent pH optima of testicular hyaluronidase preparations [34]. This notwithstanding, between pH 5 and 8 (Fig. 7, lower panel), the profile of activity of specimens from both conditioned media and cell layer extracts was similar to that of the preparation of testicular hyaluronidase, with a maximum between pH 6 and 7, thereby demonstrating that the hyaluronidase activity detected at near neutral pH does not represent the 'tail' of the pH profile of an enzyme essentially active at acid pH.

### Effect of IL-1 on the relative amounts of PH-20 in cell layers extracts and conditioned media

**Figure 8:**
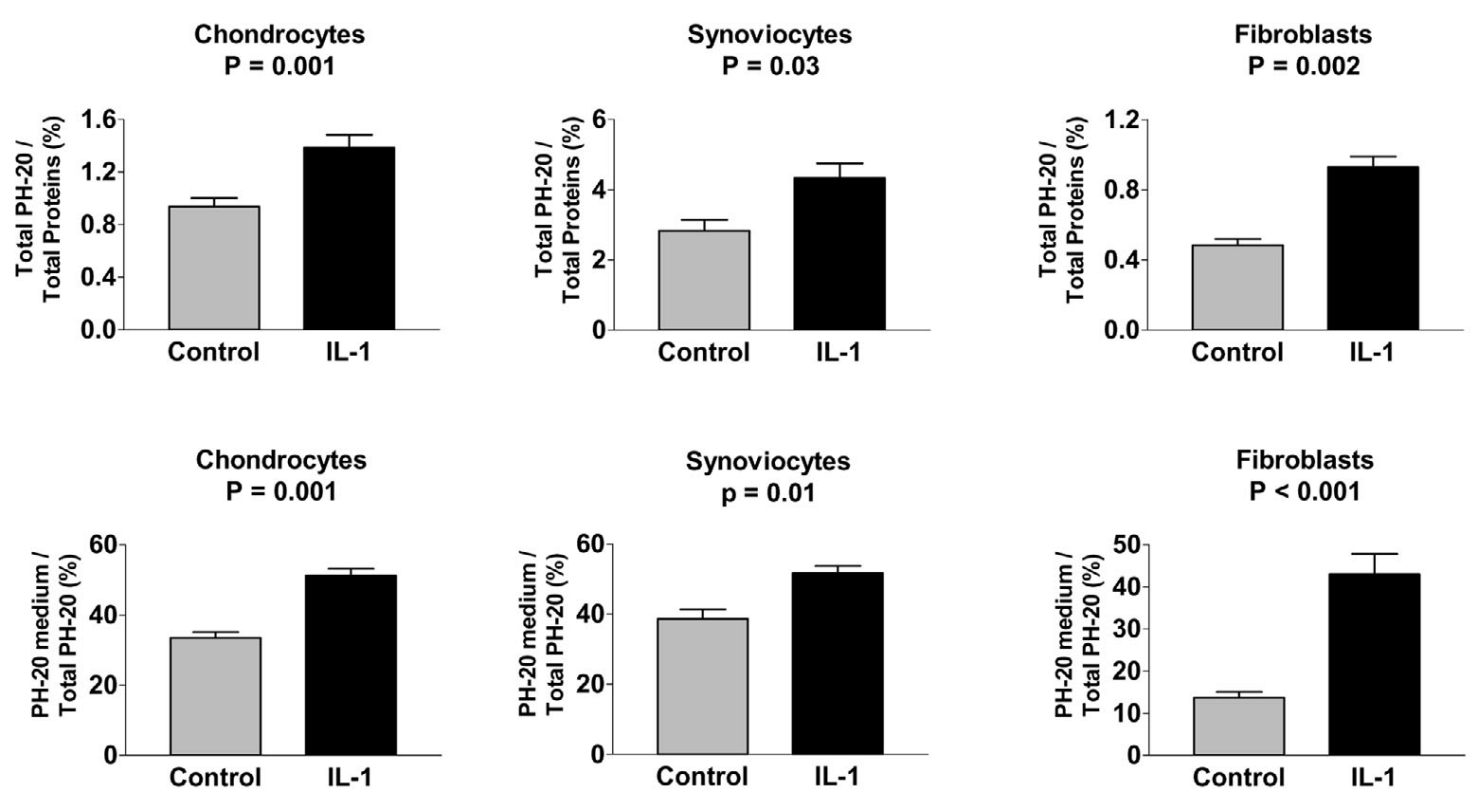
Effect of IL-1 on total PH-20 production and hyaluronidase secretion by connective tissue cell lines. Chondrocytes, synoviocytes, and fibroblast cell layers either were not stimulated or were stimulated with IL-1 at a concentration of 5 ng/ml. At the end of the stimulation period, the PH-20 proteins present in conditioned media and Triton X-100-extracted cell layers were purified by the immunoaffinity gel and quantified using the commercial BCA assay. Upper panel: total amount of PH-20 (cell layer + corresponding medium) expressed as the relative percentage of the total amount of proteins present in the Triton-X-100 cell layer extract. Lower panel: relative percentage of the total PH-20 liberated into the conditioned medium. *P *= *P *value as assessed by paired *t*-test.

Based on the above results, the immunoaffinity gel was used to evaluate to what extent IL-1 could modulate the amount of PH-20 present in the cell layers and conditioned media of chondrocytes, synoviocytes, and fibroblasts. The various cell lines were incubated for 16 hours in the absence and in the presence of IL-1 at a concentration of 5 ng/ml. For each cell line and for each condition, the amounts of PH-20 molecules immunopurified from Triton-X-100 cell layer extracts and corresponding culture media were summed and expressed as the relative percentage of the total amount of proteins present in Triton-X-100-extracted cell layers (Fig. 8, upper panels). Striking differences were observed between the three cell lines. In the absence of IL-1, fibroblasts expressed the lowest amounts of PH-20 and synoviocytes produced the highest amounts. However, stimulation with IL-1 (5 ng/ml) increased the total amount of expressed PH-20 molecules by a factor of 1.9 in fibroblasts and 1.5 in both chondrocytes and synoviocytes.

The relative amounts of PH-20 present in culture media also differed markedly from one cell line to another (Fig. 8, lower panel). For unstimulated cells, the relative amounts of PH-20 liberated into conditioned media were lowest in fibroblasts and highest in synoviocytes. However, when cells were stimulated with IL-1, the relative increase in the amount of PH-20 secreted into the culture medium were higher in fibroblasts than in either chondrocytes or synoviocytes.

## Discussion

The data reported herein strongly suggest, for the first time, that human chondrocytes, synoviocytes, and dermal fibroblasts all express, at both the mRNA and protein levels, a neutral-active hyaluronidase similar to PH-20. Further, and importantly, IL-1 enhanced PH-20 levels not only in cell layer extracts but also in culture media of the three cell lines. These observations are likely to be of great interest to both clinicians and scientists, because they shed new light on the biology of connective tissue. Indeed, this neutral-active hyaluronidase is likely to play a key role in the normal turnover of HA and in the receptor-mediated pathways of HA endocytosis. The enzyme also generates HA fragments that are highly angiogenic and are potent inducers of inflammatory cytokines. On the other hand, overexpression or uncontrolled expression of PH-20 can cause great havoc in the extracellular matrix of connective tissues, since the filamentous molecule of HA serves as a backbone upon which other macromolecules associate. Thus, like bacteria, fungi, viruses, leeches, bees, lizards, and snakes, which all use neutral-active hyaluronidase(s) to open up tissue spaces and facilitate penetration [35], spermatozoa use PH-20 to penetrate the cumulus oophorus [17], and invasive and metastatic breast cancers express high levels of PH-20 [21].

Several factors could explain why the enzyme has not been detected before in connective tissue cell lines. Northern blot analysis and the more sensitive RT-PCR technique detected the PH-20 transcript in testis, normal breast tissue, metastatic cancer cell lines, fetal and placental cDNA libraries, and murine kidneys, as well as in trace amounts in the prostate, but those studies never investigated the RNA from articular cartilage, synovium, or skin [17-21]. Two previous studies did not detect the PH-20 transcript in chondrocytes. Because of their 'gene-homology' RT-PCR approach, Flannery and colleagues [22] used reverse primers that do not match correctly the reported PH-20 cDNA sequence, whereas, although they were far from being ideal (according to the Oligo and Primer-3 programs), the primers used by Nicoll and colleagues [36] were apparently able to detect the PH-20 mRNA in testis cDNA but not in chondrocytes and fibroblasts. On the other hand, we provide strong evidence that, in contrast to Triton X-100, a detergent used to solubilize sperm PH-20 [17], octylglucoside, which was used in previous studies to solubilize the hyaluronidase activity present in chondrocytes and fibroblasts [22,28], is unable to extract PH-20 in substantial amounts from connective cell layers. Further, our data also show that detection of the neutral-active hyaluronidase at the expected molecular weight by HA substrate gel electrophoresis can be missed because the migration of the enzyme into the gel may be hampered by the interaction between, on the one hand, HA molecules of relatively high molecular weight and, on the other hand, the HA binding domain of PH-20, which is separate from the hyaluronidase domain of PH-20 [17].

HA is not an inert constituent of the extracellular matrix, but, rather, is highly metabolically active, with a half-life ranging from less than a day in the skin to about 3 weeks in articular cartilage [37,38]. As there is evidence that after a first degradation within the tissues of origin, HA is drained by the lymphatic system before being further degraded in lymph nodes, liver, and kidney [39], PH-20 is likely to contribute to the pool of HA molecules that are easily released from the tissue into the lymphatic system. There is also strong evidence that, via CD44 and/or other cell surface receptors, many cell types can bind and internalize HA, a process that, because of steric inhibition, is dependent upon the size of both HA and HA-bound macromolecules [40,41]. Since PH-20 degrades not only HA but also the chondroitin sulfate chains of the various proteoglycan molecules present in the extracellular matrix, the enzyme is likely to facilitate HA internalization, a process that does not necessarily lead to the degradation of the glycosaminoglycan to small oligosaccharides within lysosomes. Indeed, HA networks have been observed in both the cytoplasm and nucleus of several cell lines [42]. These observations and the growing list of intracellular hyaladherins, such as RHAMM, suggest that the intracellular networks of HA may regulate intracytoplasmic and intranuclear signaling events that are thought to contribute to various inflammatory processes [43].

PH-20 generates a mixture of HA oligosaccharides and fragments that may interact with various cells and produce distinct and important biological effects quite different from those induced by the native, high-molecular-weight polymer. Thus, by stimulating the proliferation and migration of vascular endothelial cells via multiple signaling pathways, HA fragments, but not high-molecular-weight HA molecules, are angiogenic [8,9]. Obviously, PH-20 produces angiogenic HA fragments, since cancer cell lines expressing this hyaluronidase induce angiogenesis in the cornea of mice whereas cancer cell lines lacking PH-20 mRNA do not [18]. Further, since PH-20 activity is enhanced by IL-1, the hyaluronidase may also contribute to the production of HA fragments that accumulate under inflammatory conditions and act as signaling molecules. Indeed, while high-molecular-weight HA molecules suppress the proliferation of synovial cells as well as the production of IL-1, prostaglandin E_2_, and matrix metalloproteinase-3 by arthritic synovium [10-12], fragmented HA molecules not only induce irreversible phenotypic and functional maturation of dendritic cells [44] but also stimulate the production of cytokines, chemokines, and nitric oxide by macrophages, an activity involving nuclear factor κB and several other transcription factors [7].

The finding that human dermis contains a neutral-active hyaluronidase suggests that depolymerization of HA can occur locally within the dermis. Accumulation of dermal HA with its associated water of hydration, as seen in urticaria and bullous skin lesions, can arise from both increased synthesis and local catabolic failures. On the other hand, PH-20-induced depolymerization of HA may contribute to the scar formation that occurs in adult wounds: fetal wounds have an extracellular matrix rich in HA of high molecular weight and heal without fibrosis, whereas the addition of hyaluronidase to the wound fluid enhances wound fibrosis [45,46]. By binding transforming growth factor β, the most critical factor involved in inflammatory fibrosis, HA helps to concentrate and to protect from proteolytic degradation this growth factor [47], which could be then released by PH-20.

Uncontrolled and/or up-regulated PH-20 activity may be very deleterious to cartilage matrix, both directly and indirectly, since even a couple of cleavages along the filamentous backbone of aggrecan aggregates dramatically reduce the viscoelastic properties of articular cartilage as well as the size of these aggregates, which become no longer effectively immobilized within the collagen network of the articular tissue. Further, the oligosaccharides produced by PH-20 have been shown to induce a dose-dependent chondrocytic chondrolysis as well as up-regulation of aggrecan synthesis and HA synthase 2 mRNA [48]. In both experimental and human osteoarthritis, the progressive reduction in the HA content of articular cartilage is believed to contribute to the apparent irreversibility of the disease process [49-51]. Because the loss of HA from cartilage explants occurs in spite of an up-regulation in HA biosynthesis [52,53], a likely explanation is that HA strands are being degraded at an accelerated rate by a hyaluronidase active at near neutral pH. On the other hand, PH-20 may be also responsible for the release of aggrecan ternary complexes made of aggrecans, link protein, and HA from cartilage matrix upon stimulation with retinoic acid, a process that persists when cartilage explants are bathed with AG3340 at concentrations that completely inhibit the collagenolytic activity present in explants as well as the enzymatic activity of both aggrecanase-1 and aggrecanase-2 [54].

The observation that IL-1 up-regulates the production of PH-20 by both chondrocytes and synoviocytes is worth noting, because enhanced expression of the enzyme may contribute to the degradation of cartilage matrix in arthritides such as rheumatoid arthritis; this contention is strengthened by the observation that proinflammatory cytokines increase the loss of HA fragments from cartilage explants [14]. On the other hand, there is evidence that proinflammatory cytokines up-regulate the levels of chondrocyte lysosomal hyaluronidases [22] as well as the expression of the CD44/HA receptor by chondrocytes [41], thereby suggesting that, within articular cartilage, HA catabolism also involves endocytosis and intracellular degradation. Although this intracellular pathway does not explain the loss of HA molecules from cartilage matrix, the intracellular and the extracellular PH-20 pathways can be complementary, since, as stated above, the degradation of high-molecular-weight HA molecules facilitates their endocytosis [40,41].

Furthermore, because the pH of synovial fluid is usually above 6 [55], PH-20 can contribute to the depolymerization of HA molecules present in this body fluid. The local production of HA fragments is likely to enhance joint degradation by producing HA fragments that, concomitantly, trigger the inflammatory reaction, and reduce dramatically the rheological properties of the joint fluid. It has been suggested that the nonsteroidal anti-inflammatory drugs, such as indomethacin, may exert a portion of their anti-inflammatory properties by inhibiting hyaluronidase and, hence, the generation of small HA fragments [56].

As PH-20 has a HA-binding domain that is distinct from its hyaluronidase domain, the molecule may also act as a HA receptor at the cell surface. Indeed, binding of HA to this distinct domain of sperm PH-20 results in thyrosine phosphorylation and an increase in intracellular calcium [17]). Glycosylphosphatidylinositol-anchored proteins involved in signaling are often associated with nonreceptor protein kinases that are bound to the cytoplasmic leaflet of the plasma membrane and are believed to regulate signal transduction [57].

## Conclusion

Our study provides strong evidence that connective tissue cells express PH-20 and that the production of this neutral-active hyaluronidase is up-regulated by IL-1. Since, besides having unique physicochemical properties, HA can modify cell behavior and plays a key role in the organization of the extracellular matrix of connective tissues, this finding may contribute new insights into the pathophysiology of several disorders including skin and joint diseases. Although the overall hyaluronidase activity detected at neutral pH was relatively low, remodeling of the extracellular matrix during wound healing, inflammatory processes, and cancer growth and metastasis are slow processes. In this context, even very slow enzymatic activities at physiological pH, operating on a time scale of hours to days rather than minutes, may suffice to create great havoc.

## Abbreviations

bp = base pairs; BSA = bovine serum albumin; DMEM = Dulbecco's modified Eagle's medium; dpm = distintegrations per minute; FCS = fetal calf serum ; HA = hyaluronan; IL-1 = interleukin-1; kb = kilobases; PBS = phosphate-buffered saline; PH-20 = sperm hyaluronidase or sperm adhesion molecule 1; RT-PCR = reverse transcriptase polymerase chain reaction; rTRUs = relative turbidity-reducing units; SSC = 0.15 M Na Cl, 0.015 M sodium citrate, pH 7.0.

## Competing interests

The author(s) declare that they have no competing interests.

## Authors' contributions

HEL played a leading role in the coordination of the study, contributed to the research protocols, participated in the interpretation of results, and prepared the manuscript. AAC conducted the *in situ *hybridization and histological studies. DHM designed the research protocols, supervised the studies, and drafted the manuscript. All authors read and approved the final manuscript.
